# The Clinical Relevance of Long Non-Coding RNAs in Cancer

**DOI:** 10.3390/cancers7040884

**Published:** 2015-10-27

**Authors:** Andreia Silva, Marc Bullock, George Calin

**Affiliations:** 1 Department of Experimental Therapeutics, The University of Texas MD Anderson Cancer Center, 1901 East Road, Houston, TX 77054, USA; andreia.silva@ineb.up.pt (A.S.); m.bullock@soton.ac.uk (M.B.); 2 Instituto de Investigação em Saúde, Universidade do Porto, Porto 4200, Portugal; 3 INEB—Institute of Biomedical Engineering, Universidade do Porto, Rua do Campo Alegre 823, Porto 4150-180, Portugal; 4 ICBAS—Instituto de Ciências Biomédicas Abel Salazar da Universidade do Porto, Rua de Jorge Viterbo Ferreira 228, Porto 4050-313, Portugal; 5 Cancer Sciences Unit, University of Southampton School of Medicine, Southampton SO16 6YD, UK; 6 Department of Surgery, University Hospital Southampton, Southampton SO16 6YD, UK; 7 Center for RNA Interference and Non-Coding RNAs, The University of Texas MD Anderson Cancer Center, 1901 East Road, Houston, TX 77054, USA

**Keywords:** biomarker, cancer diagnosis, cancer prognosis, sensitivity, specificity, circulating lncRNAs, exosomes

## Abstract

Non-coding RNAs have long been associated with cancer development and progression, and since their earliest discovery, their clinical potential in identifying and characterizing the disease has been pursued. Long non-coding (lncRNAs), a diverse class of RNA transcripts >200 nucleotides in length with limited protein coding potential, has been only modestly studied relative to other categories of non-coding RNAs. However, recent data suggests they too may be important players in cancer. In this article, we consider the value of lncRNAs in the clinical setting, and in particular their potential roles as diagnostic and prognostic markers in cancer. Furthermore, we summarize the most significant studies linking lncRNA expression in human biological samples to cancer outcomes. The diagnostic sensitivity, specificity and validity of these non-coding RNA transcripts is compared in the various biological compartments in which they have been detected including tumor tissue, whole body fluids and exosomes.

## 1. Introduction

Each cell function is controlled by highly regulated programs of gene expression, which depend on the activity of a myriad of proteins and non-protein coding RNAs (ncRNAs). The role of ncRNAs in modulating gene expression has long been recognized, and various classes of ncRNAs, with different targets and functions, have been identified [1,2]. NcRNAs can be grouped into two major classes: the small non-coding RNAs including microRNAs (miRNAs), being perhaps the most well described, and the long non-coding RNAs (lncRNAs), which were only recently discovered and comprise long intergenic RNAs, intronic RNAs, circular RNAs, competing endogenous RNA, transcribed ultra-conserved regions, antisense RNAs, and others. Crucially, one lncRNA transcript may be classified into different categories depending on the criteria which have been applied (reviewed in [3]).

LncRNAs are classically defined as RNA transcripts greater than 200 nucleotides in length, with absent or limited protein coding potential. Generally, they have fewer exons than messenger RNAs (mRNAs) and only short open reading frames can be identified, thus a minor subset of them are likely to encode small peptides. They can be transcribed from multiple locations in the genome [3], and according to the GENCODE analysis (available at www.gebcodegenes.org) of the last version of Ensembl human genome annotation (GRch38, version 23 from March 2015; [4]), 15,931 genes originating 27,817 transcripts are identified as lncRNAs [5]. In a manner similar to mRNA, many are transcribed by RNA polymerase II, with a 5’-cap and a 3’-polyadenylation, and can be variously spliced [6,7].

The analysis of lncRNAs expression in the human body revealed their presence in a variety of tissues during homeostasis, although at much lower levels than protein-coding genes. Most importantly, their expression seems to be much more tissue specific than protein coding genes [6].

**Figure 1:**
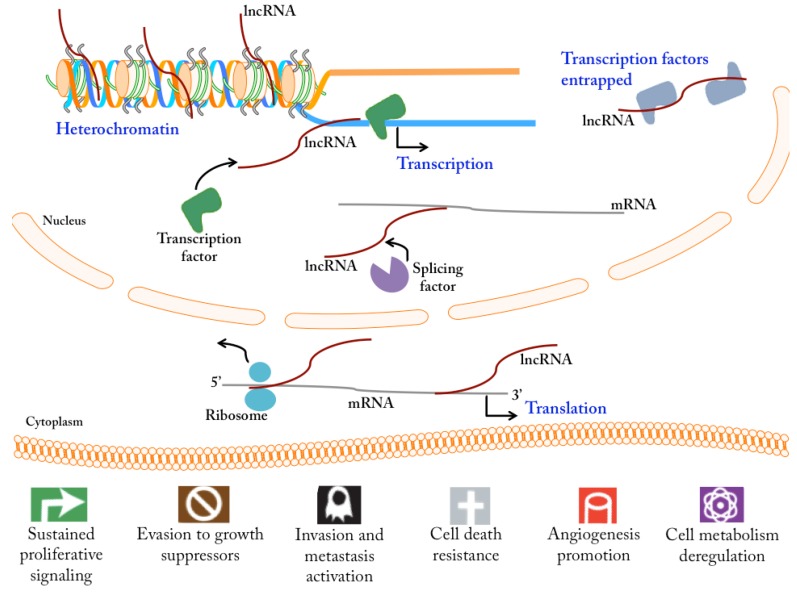
Mechanisms of action of long non-coding RNAs (lncRNAs) and implications for modulation of cancer phenotype. LncRNAs regulate gene expression by controlling chromatin condensation, promoting or inhibiting DNA transcription, influencing mRNA splicing, determining mRNA stability, and promoting or inhibiting mRNA translation into proteins. This leads to deregulated cell homeostasis, originating some of the aberrant phenotypes described as cancer hallmarks.

The majority of lncRNAs are found in the cell nucleus [6]. They have been reported to exert their function by influencing at the molecular level (Figure 1): (i) chromatin structure; (ii) transcriptional activity; (iii) mRNA stability; (iv) mRNA post-transcriptional processing; and (v) mRNA translation. At the DNA level, these transcripts are able to impair gene expression by recruiting chromatin remodeling complexes, such as the polycomb repressive complex 2, responsible for the condensation of chromatin [8]. They may either promote mRNA transcription, by co-activating transcription factors [9] and mediating gene promoter demethylation [10], or repress it, by sequestering RNA binding proteins and transcription factors [11], and by direct interaction with gene promoter regions [11]. Moreover, lncRNA may influence mRNA splicing by modulating the activity of splicing factors [12]. At the mRNA level, they may either increase the stability of the coding transcripts, preventing their degradation by perfect base-pairing [13], or decrease their stability, triggering STAU1-mediated mRNA degradation [14]. Lastly, lncRNAs may prevent mRNA translation through direct interaction with the transcripts, impairing ribosome binding [15], or conversely, promote it, in a process driven by a subset of antisense lncRNAs that overlap with the target mRNA at the 5’ end, leading to a higher association of polysomes and mRNA [16].

The basal expression of lncRNAs in many tissues has been shown to be important in various biological homeostatic processes, including gene imprinting and dosage-compensation [17], cell differentiation and organogenesis [18,19,20] and immune response modulation [21], among others. On the other hand, there is a strong link between the deregulated expression of lncRNAs and the development of disease. Indeed, aberrant lncRNAs expression has been found in neurodegenerative disorders [22], cardiovascular diseases [23], muscular dystrophies [19], diabetes and obesity [24], as well as cancer biology.

In this article, we focus on the aberrant expression of lncRNA in cancer. We present the main lncRNA candidates found to be deregulated in cancer through the analysis of patient biological material. Consequently, the validity of using these deregulated lncRNAs as biomarkers for cancer diagnosis and patients monitoring is discussed.

## 2. lncRNAs as Biomarkers for Cancer Diagnosis and Prognosis

### 2.1. Functional Role of lncRNAs in Cancer

Tumorigenesis is associated with an overall deregulation of different biomolecules, including lncRNAs. This deregulation of lncRNAs may be observed not only at their intracellular/tissue levels but also at their levels on extracellular body fluids. In order to understand the biological effects of an aberrant expression pattern of lncRNAs at the cellular level, several *in vitro* and *in vivo* studies have relied on artificial up-regulation and down-regulation of specific lncRNAs by different techniques. With this approach, deregulated expression of different lncRNAs, as detected either in tumor tissues and/or in biological fluids, have been highly correlated with cell functions and features defined as the hallmark processes of cancer (Figure 1).

One of the most studied lncRNAs, MALAT1 (Metastasis Associated Lung Adenocarcinoma Transcript 1) promotes tumor growth by regulating cell cycle. Down-regulation of MALAT1 *in vitro* in cell lines of different cancer types (*i.e.*, breast cancer, colorectal cancer, esophageal squamous cell carcinoma, renal cell carcinoma, and others) leads to reduced cell proliferation by cell cycle arrest at the G2/M phase, and to cell apoptosis [25,26,27,28]. This ultimately results in the reduced capacity of cells to invade and migrate. Similarly, down-regulation of MALAT1 in cell lines of different tumor types inhibits tumor growth in *in vivo* models using tumor xenografts in nude mice [27]. These findings are supported by complementary MALAT1 overexpression studies [26]. MALAT1 has been shown to promote epithelial-to-mesenchymal transition [28] and appears to regulate angiogenesis [29], further supporting its potential role during cancer progression.

The lncRNA HOTAIR (HOX transcript antisense RNA) has also been associated with stimulation of cellular proliferation, but appears to have more potent effects on cellular migration and invasion. Furthermore, its expression is highly correlated with the presence of metastasis in clinical samples. Overexpression of HOTAIR in breast [30], gastric [31] and lung [32] cancer cell lines, stimulates an increased invasive capacity *in vitro*. More importantly however, overexpression of HOTAIR in non-metastatic cell lines leads to a high degree of metastization *in vivo* [30]. As expected, HOTAIR is a strong inducer of epithelial-to-mesenchymal transition [33].

Deregulated lncRNAs are also implicated in the evasion of growth suppression signals in cancer. For example, GAS5 (Growth Arrest-Specific 5), commonly down-regulated in multiple cancer types, is actually an inducer of apoptosis, thus limiting cell proliferation when expressed at homeostatic levels [34].

Lastly, regulation of metabolism in cancer cells is also influenced by lncRNAs. The prostate cancer-associated lncRNA PCGEM1 was recently shown to promote glucose uptake in prostate cancer cell lines, conferring these cells an overall metabolic advantage by regulating at the transcriptional level, not only glucose metabolism, but also glutamine metabolism, the pentose phosphate catabolic pathway, the tricarboxylic acid cycle and fatty acids and nucleotides synthesis pathways [35].

### 2.2. Expression of lncRNAs in Tumor Tissue

Several lncRNAs have been shown to be deregulated in tumor tissue of various cancer types (Table 1). Whilst some appear to be deregulated in all cancers regardless of histopathological type, others demonstrate high levels of tissue specificity, highlighting a potential role for them as biomarkers for early cancer diagnosis, disease evolution or poor prognosis outcome.

MALAT1 and HOTAIR are both examples of lncRNAs which are deregulated in the majority of cancers. MALAT1 overexpression in tumor tissue has been particularly linked to lung cancer, colorectal cancer, gastric cancer, and hepatocellular carcinoma (HCC). Given this global pattern of deregulation in cancer, the biomarker potential of MALAT1 lies perhaps more in its prognostic rather than diagnostic application, correlating as it does with a poor outcome for patients with cancer [36]. For instance, Zheng *et al.*, showed that MALAT1 expression in colorectal cancer tissue (stage II and III) significantly correlates inversely with disease-free survival and overall survival, where patients with the highest levels of MALAT1 have a probable five-year disease-free survival and overall survival of 48% and 67% respectively, compared with 67% and 85% in patients with low expression levels [37].

Similar observations have been extended to HOTAIR, the contribution of which appears particularly relevant in breast cancer, lung cancer and cancers of the digestive tract [38]. As is the case for MALAT1, HOTAIR is considered most valuable as a prognostic rather than diagnostic biomarker, and in the identification of metastatic potential in particular [39]. Indeed, high levels of this lncRNA have been linked to poor survival outcomes for patients with colorectal cancer, which have a probable five-year overall survival of only approximately 55%, compared to 80% in patients that express lower levels of the transcript in the tumor specimen [40]. Similarly, in a previous study in breast cancer, higher levels of HOTAIR were associated with reduced patient survival and, more interestingly, with a decrease in the probability of metastasis-free survival of nearly 50%, compared to patients with lower expression of the transcript [30], which supports the role of this lncRNA as a metastization biomarker.

At a more tissue-specific level, PCA3 (Prostate Cancer Antigen 3 lncRNA; also referred to as DD3) has been shown to be up-regulated in prostate tumor tissue *versus* normal/non-malignant tissue in multiple studies [41,42]. One of the first studies published on this subject suggested that the performance of PCA3 as a diagnostic biomarker was associated with an area under the curve (AUC) in a receiver operating characteristic curve (ROC) analysis of 0.98 [43]. Furthermore, Bussemakers *et al.* [41] demonstrated that PCA3 expression is highly specific for prostate tumors, being undetectable in other types of tumors.

Similarly, PCGEM1 is specifically expressed in the prostate, and up-regulated in tumor tissue of prostatic origin compared with matched normal prostate tissue samples [44]. However, the biomarker potential of this lncRNA for prostate cancer diagnosis and prognosis has subsequently been questioned, as no association has been found between high levels of PCGEM1 and prostate cancer-specific mortality [45].

The linc-RNA UCA1 (urothelial carcinoma associated 1) is an lncRNA identified as a potential biomarker for bladder cancer [46], with higher levels detected in tumor tissue and the potential to discriminate between bladder/urothelial cancer and cancers of other anatomical origins. In addition, it can be detected in the cellular sediment of urothelial cancer patients’ urine, allowing disease diagnosis with a sensitivity of 80.9%. Importantly, it allows the distinction of bladder cancer from other diseases related with the urinary tract, such as neurogenic bladder, renal cell carcinoma, upper urinary tract restriction or reflux, among others, with an overall specificity of 91.8%. An ROC analysis of UCA1 detection lead to an AUC equal to 0.882 suggesting reasonable efficacy of this lncRNA in bladder/urothelial cancer diagnosis [46]. Work by Srivastava *et al.*, showed a similar result with even higher levels of the transcript being detected in urine with progressive tumor stage [47].

**Table 1:** Validity of circulating long non-coding RNAs as biomarkers for diagnosis of different types of cancer. Exemplificative data from most recently published studies is presented.

Cancer Type	lncRNA	Biological Sample	Fold-Change to Normal Control	Number of Patients	Specificity	Sensitivity	AUC	Ref.
Lung cancer	MALAT1	Peripheral blood cells	↓ 0.30	45	96%	56%	0.79	[48]
Colorectal cancer	HOTAIR	Peripheral blood cells	↑ 5.22	84	92.5%	67%	0.87	[49]
Prostate cancer	PCA3	Urine	n/a	3245	75%	62%	0.75	[50]
			↑ 2.58 *	407	60.1%	94.9%	0.87	[51]
			↑ n/a	3073	75%	53%	0.69	[52]
	MALAT1	Plasma	↑ n/a	87	58.6%	84.8%	0.84	[53]
Hepatocellular carcinoma	RP11-160H22.5 XLOC_014172 LOC149086	Plasma	↑ 2.5 ↑ 67.7 ↑ 4.6	467	73%	82%	0.896	[54]
Bladder cancer	UCA1	Urine	↑ n/a	94	91.8%	80.9%	0.88	[46]
			↑ 32.9	117	79.7%	79.5%	0.86	[47]
Gastric cancer	AA174084 AA174084	Tissue Gastric juice	↓ 3.18 ↑ n/a	134 39	73% 93%	57% 46%	0.68 0.85	[55]
	LINC00152	Plasma/plasma exosomes	↑ n/a	79	85.2%	48.1%	0.66	[56]

Arrows represent the up-regulation (↑) or down-regulation (↓) of the transcript. * Fold-change of PCA3 score, as determined by PROGENSA PCA3 assay. n/a, not available, since data is presented only in graphical format in the original report.

In hepatocellular carcinoma, the lncRNA HULC (Highly Up-regulated in Liver Cancer) was proposed as a diagnosis biomarker, as it is up-regulated in liver tumor relatively to hepatic tissue from healthy individuals. An ROC analysis of HULC expression in liver tissue to distinguish tumor from healthy tissue resulted in an AUC of 0.86. Further, HULC is expressed at higher levels in tumors with a higher Edmondson grade classification, correlating with disease aggressiveness, and thus supporting a potential prognosis biomarker role [57].

In a pioneer study [55], Shao *et al.*, investigated the biomarker potential of the lncRNA AA174084 for gastric cancer detection, and found the expression of the transcript was nearly 3.18 times lower in primary gastric tumor tissues of 71% of the patients evaluated, compared with the expression levels in the matched normal gastric tissue. Furthermore, an inverse relationship between expression of this transcript and the aggressiveness of the gastric mucosal lesions was identified. Indeed, AA174084 expression in gastric tissue permitted the distinction of cancer from benign histology with a sensitivity of 57% and a specificity of 73%. Notably, however, in ROC analysis, AA174084 expression as a diagnostic marker was associated with an AUC of only 0.676, which is insufficiently discriminatory for use in the clinical setting.

In a number of studies, cancer relevant lncRNAs have been detected not only in primary tumor tissue but also in the peripheral fluids of patients with cancer.

For example, MALAT1 has been detected in the blood of patients with non-small cell lung cancer [48], and HOTAIR in colorectal cancer patients [49]. This research theme holds great promise both for diagnostician and patient and will be explored in more detail in the following section.

### 2.3. Detection of Cell-Free lncRNAs in Body Fluids

Patterns of lncRNA deregulation in primary tumor tissues are mirrored in various bodily fluids, including plasma and urine, as summarized in Table 1. This presents an opportunity to develop lncRNA based biomarker tools which are convenient, minimally invasive, and likely to be better tolerated by patients than conventional tissue biopsies.

The potential of circulating PCA3 as a biomarker for prostate cancer has been explored in several studies, which have quantified transcript expression in patients’ urine. A meta-analysis of several of these studies determined the validity of PCA3 levels in urine for prostate cancer diagnosis, with a summary sensitivity of 62% and specificity of 75%. In an ROC analysis, this translated to an AUC of 0.75, further supporting PCA3 as a reasonable marker for prostate cancer diagnosis [50]. Similar results were obtained in a second independent meta-analysis [58], in which the sensitivity and specificity for prostate cancer diagnosis was calculated as 57% and 71%, respectively, and the AUC as 0.7118 [58]. Circulating PCA3 has also prognostic value for the evaluation of prostate cancer disease evolution, since its levels correlate with tumor aggressiveness as classified by Gleason score [51,52]. In addition, in prostate cancer patients, fragments from different regions of MALAT1 transcript were detected in plasma at higher copy number than in non-prostate cancer patients. This lncRNA was proposed as a biomarker for prostate cancer diagnosis with a sensitivity and specificity of 58.6% and 84.8%, respectively, corresponding to a promising AUC of 0.836 [53].

In the same way, evaluation of cell-free HULC levels has been assessed for the diagnosis of HCC. In fact, in the study by Xie *et al.*, HULC was detectable at high levels in the plasma of 63% of the hepatocellular carcinoma patients enrolled in screening. Unfortunately, no data was offered regarding specificity and sensitivity of HULC expression in plasma for the diagnosis of HCC patients in this study [57]. On the other hand, other lncRNAs less commonly studied were recently proposed for hepatocellular carcinoma diagnosis. Tang *et al.* identified an up-regulation of the transcripts RP11-160H22.5, XLOC_014172 and LOC149086 in the plasma of HCC patients relative to cancer-free controls. The combination of the three lncRNAs has better scores for HCC diagnosis comparing to each individual lncRNA, corresponding to a merged AUC of 0.896, with a sensitivity of 82% and specificity of 73% [54]. Interestingly, lncRNAs XLOC_014172 and LOC149086 also have a prognostic value for metastasis prediction, distinguishing HCC patients with metastasis from patients without, with a sensitivity and specificity of 91% and 90%, respectively (AUC for the combined lncRNAs of 0.934) [54]. Additionally, another study identified lncRNA-AF085935 in serum as a potential biomarker for HCC diagnosis, allowing not only a distinction of HCC patients from healthy control individuals but also of HCC patients from hepatitis B-infected patients, corresponding to an AUC of 0.96 and of 0.86, respectively [59].

So far, plasma/serum and urine are the bodily fluids that have been most commonly used for lncRNA profiling in cancer patients; however, other fluids have also been tested. For instance, expression of the lncRNA AA174084 was evaluated in gastric juice for the diagnosis of gastric cancer patients [55]. In this fluid, the levels of the lncRNA were significantly higher than in healthy individuals and patients with other gastric mucosa lesions, corresponding to an AUC of 0.848 for gastric cancer diagnosis. AA174084 in digestive fluids presented in this way as a robust biomarker, allowing the diagnosis of the disease with a sensitivity of only 0.46 but a specificity of 0.93. Most interestingly, the levels of the same lncRNA in the plasma could not distinguish gastric cancer patients from healthy individuals.

Focusing on less commonly analyzed body fluids, Tang *et al.*, were able to detect by qPCR HOTAIR and MALAT-1 in salivary samples of nine patients with oral squamous cell carcinoma, although a threshold of Cq < 40 cycles was adopted for positive samples classification [60]. In this way, more robust studies exploring the detection of lncRNAs in body fluids other than plasma and urine, and the real relevance they might have for cancer diagnosis and prognosis, are still missing.

Most of the studies published to date have screened for lncRNA expression in whole plasma/serum or whole urine. Nevertheless, evidence exists that at least part of the circulating lncRNA transcriptome is present in “subcompartments” of those biological samples, such as extracellular vesicles released by cells. In 2009, a proof-of-concept study showed the presence of PCA3 transcripts within exosomes [61]. Later, an attempt to profile the genetic material enclosed within exosomes isolated from plasma of healthy blood donors demonstrated that lncRNAs account for 3.36% of total exosomal RNA content [62]. Interestingly, the ratio of different RNA transcripts within exosomes appears to differ from their cells of origin, suggesting that lncRNA are actively loaded in a controlled manner into these vesicles [63].

Exosomes are nanometric lipidic vesicles secreted by cells and which mediate cell-to-cell communication. Enclosed within a lipid bilayer, exosomes carry proteins and genetic material, including DNA, mRNA, miRNA and lncRNA, which are transferred to specific target cells. Interestingly, the loading of exosomes is dependent on the activity of the parental cell, and they can provide a snapshot of cellular and tissue physiology [64]. In the last few years, the content of exosomes, namely their miRNAs, have been extensively studied, with these vesicles being particularly explored as carriers of miRNAs biomarkers for various diseases [65]. In fact, different exosomal miRNAs are already well accepted as biomarkers for diagnosis and prognosis of different types of cancer [66]. Recently the same goals have been pursued for lncRNAs.

Like other transcripts contained within exosomes, lncRNAs may be transferred between cells and have functional relevance within recipient cells [67]. This work by Takahashi *et al.* demonstrated that the long intergenic non-coding RNA ROR (linc-ROR) is an effector of HepG2 liver hepatocellular carcinoma cell line chemoresistance to sorafenib treatment, and is present in exosomes released by these cells. Furthermore, chemoresistance may be transferred to other HepG2 cells upon stimulation with exosomes derived from chemoresistant cells, supporting the functional and pathological transfer of linc-ROR to recipient cells. In addition, a change in the content of lncRNAs in the exosomes released by these cells is detected upon cell stimulation with TGF-β *in vitro*. This further supports the recognition of exosomes as cells snapshots, reflecting their physiology and phenotype, and thus as a great resource for potential cancer biomarkers identification.

In a new study [68], exosomal lncRNAs are further implicated in cancer development, namely through the modulation of cancer microenvironment. Here, it has been demonstrated that CD90^+^ hepatocellular cancer cells derived from the Huh7 cell line secrete exosomes containing different types of lncRNAs, including HOTAIR, HULC, linc-ROR and H19. Upon co-culture *in vitro*, endothelial cells rapidly internalized these exosomes which triggered cell re-organization into tubular-like structures and an increase in VEGF/VEGF-R1 mRNA levels, concordant with a pro-angiogenic effect of the exosomes. In addition, exosomes promoted an increase of adhesion molecules previously described as players in extravasation processes on endothelial cells surface, thus further implicating exosomes released by CD90^+^ hepatocellular cancer cells in metastasis. The artificial over-expression of linc-ROR on endothelial cells supported the effects of exosomes being mediated by exosomal H19.

In other work [69], elevated levels of exosomal lncRNA-p21 were shown to distinguish prostate cancer patients from patients with benign prostatic hyperplasia, although it was not clear if pure populations of exosomes were isolated for this analysis, or whether circulating RNA-protein complexes and other cell-secreted vesicles had been simultaneous analyzed.

Li *et al.* [56] analyzed the levels of the lncRNA LINC00152 in plasma and plasma-derived exosomes of gastric cancer patients, and found no statistical significant difference in expression between the two sample types. This suggests that at least the majority of LINC00152 in plasma is derived from exosomes. Li further demonstrated that this exosomal lncRNA supports a diagnosis of gastric cancer with a sensitivity of 48.1% and a specificity of 85.2%, with corresponding AUC from ROC analysis of 0.66.

Lastly, exosomal lncRNAs have also been shown to have prognostic potential in cancer. Indeed, HOTAIR, found in exosomes isolated from serum of laryngeal squamous cell carcinoma patients, is elevated in samples from patients with lymph node metastasis relative to patients without lymph node metastasis, increasing also with progressive disease stage [70].

Overall, circulating lncRNAs are considered suitable biomarkers for cancer diagnosis and prognosis, not only because of the convenience of biological samples collection for the patient but also because they are quite stable RNA molecules [71] that can be detected by common techniques, such as quantitative real-time PCR, microarray hybridization and sequencing. Nevertheless, their absolute concentration in body fluids is usually low, frequently requiring an RNA amplification step prior to their analysis, and their integrity may be compromised by technical procedures related to biological sample collection and preservation, impacting their accurate quantification [72]. In addition, the mechanism of lncRNAs secretion is not yet fully unraveled, and thus the levels of circulating lncRNAs may be affected by other concomitant biological changes besides tumorigenesis. For cancer diagnosis and prognosis, these drawbacks may be minimized by the combined analysis of candidate lncRNAs together with other biomarkers previously established, such as proteins and miRNAs [72]. One of the most elucidative examples of this approach is the analysis of circulating PCA3 lncRNA and Prostate-Specific Antigen (PSA) protein for prostate cancer diagnosis [73]. Thus, lncRNAs have great potential as cancer biomarkers, either alone or analyzed together with other markers, ultimately contributing to more accurate diagnosis and prognosis of disease evolution.

### 2.4. Discovery of lncRNAs as Cancer Biomarkers: Implications in Therapeutics

The discovery of lncRNA deregulation in cancer, along with a high tissue specific expression pattern, turned them into new potential targets for the development of anti-cancer therapies. Indeed, several approaches have been proposed to reestablish the homeostatic levels of lncRNAs.

One of the most explored methods to inhibit up-regulated oncogenic lncRNAs is the delivery of small interfering RNAs (siRNAs) to target cells. These siRNAs are complementary to their target lncRNAs, inducing their degradation in RISC (RNA-induced silencing) complex, and consequently controlling the activity of these transcripts by decreasing their levels [74]. Another similar approach is based on the use of longer antisense oligonucleotides complementary to the target lncRNAs, which promote their degradation by RNase H [75]. On the other hand, the expression of lncRNA may be induced by common gene therapy strategies.

Taking into account the multiple mechanisms of action of lncRNAs, namely their interaction with proteins involved in chromatin organization, transcription and translation, additional therapeutic strategies can be developed that reduce lncRNA aberrant function by targeting their interaction with these proteins. Small molecules are an example of inhibitors capable of disrupting lncRNA-protein interactions, re-establishing the normal activity of these proteins [76].

Conceptually, therapeutic strategies similar to the ones already attempted to regulate the expression of protein-coding genes and miRNAs could be applied to the lncRNA field; nevertheless, the knowledge regarding the lncRNA functional network is still very limited, preventing early translational outcomes. Indeed, to date, only one phase 1/2a preliminary clinical trial is completed related to lncRNAs application for cancer therapeutics. In this study, a DNA vector expressing the diphtheria-toxin gene under the control of regulatory sequences of the lncRNA H19 was administered intratumorally in unresectable pancreatic cancer patients, envisaging tumor reduction for further complementary therapies [77].

## 3. Conclusions

In an attempt to find molecular signatures that allow more robust cancer diagnosis, many studies have focused on the comparison of biological samples from cancer patients and healthy individuals. Along the way, the ncRNA transcriptome has attracted increasing attention. These transcripts have biological activity *per se* and, unlike mRNA, they are not dependent on translation into effector proteins, making it more likely that ncRNA levels directly and more accurately correlate with defined cancer phenotypes [78].

MiRNAs are increasingly recognized as cancer biomarkers, in particular cell-free miRNAs circulating in body fluids [79]. In comparison, the development of lncRNA biomarkers is substantially retarded. The first studies published on deregulated lncRNAs in cancer were based on the evaluation of expression levels in tumor tissue. From a clinical point of view, this represented a new paradigm for patient prognosis and the guidance of therapy. However, despite reasonable correlation between lncRNA expression and patient diagnosis and/or tumor stage, this approach still required invasive procedures in order to collect a tissue specimen. This limitation prompted analysis of cell-free lncRNAs, present within various bodily fluids, or enclosed on lipidic vesicles secreted by cells. Plasma/serum and urine are the body fluids which have most commonly been analyzed for lncRNA expression. This approach has been particularly useful in the field of prostate cancer. Interestingly, both tumor tissue and cell-free lncRNAs appear to permit prostate cancer diagnosis with a high degree of specificity, as it seems lncRNA expression is more tissue-specific than other ncRNAs and mRNAs. However, the sensitivity of detection using lncRNAs is rather low, and greatly dependent on the technical methods available for lncRNAs analysis.

With recent improvements in technology associated with RNA analysis [80], higher sensitivity may in the future be achieved and, in time, lncRNAs may be recognized as cancer biomarkers with utility in the clinical setting. In order to reach this point, further studies to include larger patient cohorts will also be required.

Given the strong correlations that have been established between deregulated lncRNAs expression and cancer development and disease prognosis, therapy strategies targeting these transcripts have also been postulated. In one particularly promising field of research, it has been proposed that lncRNAs regulate miRNA expression. Thus, cancer therapies which target both miRNA and lncRNA expression may in future become a reality [81].
